# Fungal Infection Intensity and Zoospore Output of *Atelopus zeteki*, a Potential Acute Chytrid Supershedder

**DOI:** 10.1371/journal.pone.0093356

**Published:** 2014-03-27

**Authors:** Graziella V. DiRenzo, Penny F. Langhammer, Kelly R. Zamudio, Karen R. Lips

**Affiliations:** 1 Department of Biology, University of Maryland, College Park, Maryland, United States of America; 2 School of Life Sciences, Arizona State University, Tempe, Arizona, United States of America; 3 Department of Ecology and Evolutionary Biology, Cornell University, Ithaca, New York, United States of America; Imperial College Faculty of Medicine, United Kingdom

## Abstract

Amphibians vary in their response to infection by the amphibian-killing chytrid fungus, *Batrachochytrium dendrobatidis* (*Bd*). Highly susceptible species are the first to decline and/or disappear once *Bd* arrives at a site. These competent hosts likely facilitate *Bd* proliferation because of ineffective innate and/or acquired immune defenses. We show that *Atelopus zeteki*, a highly susceptible species that has undergone substantial population declines throughout its range, rapidly and exponentially increases skin *Bd* infection intensity, achieving intensities that are several orders of magnitude greater than most other species reported. We experimentally infected individuals that were never exposed to *Bd* (n = 5) or previously exposed to an attenuated *Bd* strain (JEL427-P39; n = 3). Within seven days post-inoculation, the average *Bd* infection intensity was 18,213 zoospores (SE: 9,010; range: 0 to 66,928). Both average *Bd* infection intensity and zoospore output (i.e., the number of zoospores released per minute by an infected individual) increased exponentially until time of death (*t_50_* = 7.018, *p*<0.001, *t_46_* = 3.164, *p* = 0.001, respectively). Mean *Bd* infection intensity and zoospore output at death were 4,334,422 zoospores (SE: 1,236,431) and 23.55 zoospores per minute (SE: 22.78), respectively, with as many as 9,584,158 zoospores on a single individual. The daily percent increases in *Bd* infection intensity and zoospore output were 35.4% (SE: 0.05) and 13.1% (SE: 0.04), respectively. We also found that *Bd* infection intensity and zoospore output were positively correlated (*t_43_* = 3.926, *p*<0.001). All animals died between 22 and 33 days post-inoculation (mean: 28.88; SE: 1.58). Prior *Bd* infection had no effect on survival, *Bd* infection intensity, or zoospore output. We conclude that *A. zeteki*, a highly susceptible amphibian species, may be an acute supershedder. Our results can inform epidemiological models to estimate *Bd* outbreak probability, especially as they relate to reintroduction programs.

## Introduction

Differences in amphibian susceptibility to *Batrochochytrium dendrobatidis* (*Bd*) infection were evident since the pathogen was first described [1], [2]. Species-specific responses to infection range from tolerant [3],[4] or resistant [5] to highly susceptible [6], [7], suggesting that a subset of species can disproportionately affect pathogen spread and disease transmission [8], [9]. Yet, we know relatively little about contact rates, infectivity, and zoospore output of *Bd*'s amphibian hosts in either the field or laboratory.

Differences in species transmission rates can cause variations in pathogen spread and dispersal in the wild [10]–[12]. One illustration of the potential effects of variable inter-specific interactions are superspreaders [8], individuals or species responsible for a greater than average number of secondary infections [8], [12], [13]. Superspreading occurs under two scenarios: (1) supercontacters transmit more disease by making more contacts in the population per individual, or (2) supershedders transmit more disease per contact (reviewed by [14]). To date, the primary evidence for superspreading stems from supercontacters (e.g., [15]–[17]); but growing evidence shows that species vary consistently in pathogen infection intensities (e.g., [18], [19]), especially in the amphibian-*Bd* system (e.g., [20], [21]).

An amphibian's *Bd* infection intensity likely determines its infectivity (i.e., an individual's ability to infect another individual) and survival time [6], [22], [23]. A host's *Bd* infection intensity increases via reinfection by zoospores released onto the surface of the skin or by infection from zoospores in the environment. Quantifying host-specific *Bd* zoospore output, the number of zoospores released per minute by an infected individual [4], is critical to understanding differences in infectivity across species and species-specific contributions to the environmental zoospore pool.

Highly susceptible amphibian species typically die at high *Bd* infection intensities (e.g., [7], [22]), suggesting that highly susceptible species may act as supershedders for a short period of time. In several cases across Central America [24], [25], *Bd* has caused the decline and extirpation of harlequin frog (genus: *Atelopus*) populations. Of the 113 *Atelopus* species, as many as 30 species have been declared Extinct in the Wild [24], and according to the IUCN, 80% of *Atelopus* species are Critically Endangered and 70% have declining populations. *Atelopus* experience rapid widespread population declines upon *Bd* site invasion, demonstrating high susceptibility. Here, we refer to *Atelopus* as a candidate acute supershedder to better describe the phenomena of high susceptibility and pathogen shedding.

Our goals in this study were to: (1) quantify *Bd* infection intensity and zoospore output of *Atelopus zeteki*, (2) determine the daily percent increase of *Bd* infection intensity and zoospore output on *A. zeteki*, and (3) determine if prior *Bd* exposure affects infection intensity and zoospore output. Our results are important in understanding species and community responses to *Bd* invasion and are relevant to future reintroduction programs.

## Methods

### Ethics statement

Our research strictly followed the guidelines of and was approved by the University of Maryland Institute for Animal Care and Use Committee (protocol #R-12-98) and the Maryland Zoo in Baltimore Institutional Animal Care and Use Committee.

### Experimental procedures

We obtained 13 captive-bred *A. zeteki* individuals, 15 months post-metamorphosis, used in an earlier *Bd* experiment [26]. Ten animals were uninfected controls, and three were previously inoculated with JEL 427-P39 23 weeks before the start of our experiment. During the course of the earlier experiment [26], individuals were swabbed once every two weeks for 130 days. One individual consistently tested *Bd* negative for the duration of that experiment. The other two individuals tested *Bd* positive three and four times, respectively. The last swabbing event was five weeks before the start of our experiment where two of the three individuals were mildly infected.

We matched individuals by weight into two groups of five. We found no difference in weight between the infected and control groups at the start of the experiment (*p*>0.05). The three individuals previously exposed to *Bd* strain JEL 427-P39 were assigned to the infected treatment. All individuals were sexed by examination for eggs, ovaries, or testicles at time of death (12 female and 1 male). The single male had been placed in the control treatment.

Animals were housed in plastic boxes filled with sphagnum moss, a hide, and a water dish, in a laboratory maintained at 21–22°C with a 12∶12 light∶ dark photoperiod. We replaced all housing materials every seven days, changed water dishes every three days, fed frogs vitamin-dusted crickets or fruit flies (*Drosophila melanogaster*) *ad libitum* every three days, and misted terraria daily. We monitored individuals daily for clinical symptoms of *Bd* and euthanized all individuals once they lost righting abilities by applying Benzocaine 20% gel to the venter. All control individuals were euthanized when the last infected individual was euthanized.

We inoculated individuals with *Bd* strain JEL 423, a member of the hypervirulent *Bd*GPL lineage, originally isolated from an infected *Hylomantis lemur* during the epidemic at El Copé, Panama in 2004 [27]. We grew *Bd* strain JEL 423 on 1% tryptone agar plates for seven days, flooded plates with 1% trypone broth, filtered the liquid to obtain a pure zoospore stock solution, and diluted the pure stock solution with water to achieve the desired concentration [26]. We individually inoculated the eight infected treatment frogs with 30,000 *Bd* zoospores for 10 hours. The five control individuals were exposed to a sham solution of water and <1% tryptone broth, roughly the same amount that had been used for the *Bd* treatment minus the zoospores, for the same period.

We used a fresh pair of latex powder-free gloves when handling each individual. We followed the swabbing protocol of Hyatt et al. [28]. Immediately post-swabbing, we individually soaked each frog in 50 mL of distilled water for 15 minutes and added 50 µL of bovine serum albumen (BSA) to the water solution after removing each frog [4]. We immediately filtered the solution using a 60 mL sterile syringe and 0.45 µm filter for each sample. Filters were plugged with syringe caps and stored in a 4°C refrigerator. Swabbing individuals before soaking could reduce the number of *Bd* zoospores estimated from the soak, thus our estimates are minimum zoospore output estimates.

We swabbed and soaked all individuals starting on day seven post-inoculation, thereafter every three to four days, and immediately prior to euthanasia. We extracted DNA from samples using PrepMan Ultra and analyzed samples using the standard real-time quantitative polymerase chain reaction assay [28], [29]. *Bd* infection intensity was defined as the number of *Bd* genomic equivalents detected on a single swab [7]. We categorized individuals as *Bd*-positive when *Bd* infection intensity was greater than or equal to one zoospore genomic equivalent [30].

We performed all statistical analyses in R [31]. We modeled the change in *Bd* infection intensity (*N*) with respect to time (*t*) using dN/dt = y_0_e^rt^, where y_0_ is the initial infection intensity, r is the daily rate of increase of infection intensity, and t is time in days. We used the same equation to model the change in zoospore output with respect to time. To calculate parameter estimates, we fitted two linear mixed models with a first order autoregressive correlation term to ln transformed response variables (i.e., *Bd* infection intensity and zoospore output; package *nlme*, [32]). We included prior infection history as an independent variable to determine if prior *Bd* exposure affected either response variable. We used AIC to compare model fit.

To determine if *Bd* infection intensity and zoospore output were correlated, we used a generalized linear mixed model with a first order autoregressive correlation term and a lognormal error distribution. To determine if survival curves of frogs with different infection histories differed, we used a logrank-test (package *survival*, [33]).

## Results

**Figure 1:**
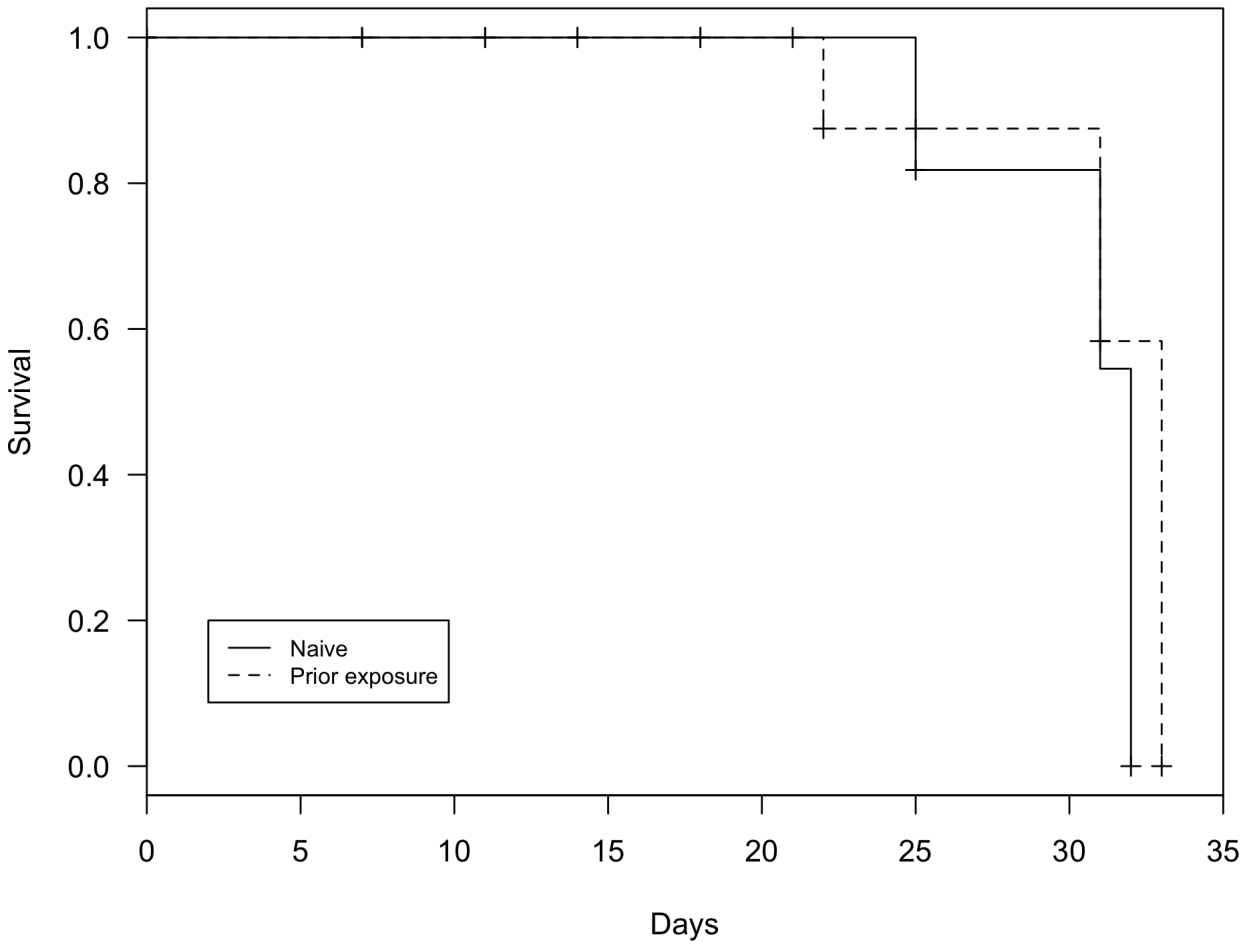
Survival curves of *Atelopus zeteki* with (*n* = 3) and without (*n* = 5) prior *Bd* exposure (log-rank test: χ^2^ = 0.7, *p* = 0.40).

All frogs exposed to *Bd* lost righting abilities and were euthanized within 33 days post-inoculation (Figure 1; 100% mortality, mean: 28.88 days, SE: 1.58). All control animals tested negative at all sampling events, and no control animal experienced mortality during the course of the experiment.

At time of death, infected frogs had an average *Bd* infection intensity of 4,334,422 zoospores (SE: 1,156,576; range = 520,436 to 9,584,158) and an average zoospore output of 23.55 zoospores per minute (SE: 22.78; range = 0.00 to 172.61; Table 1).

**Table 1:** Summary of *Atelopus zeteki* infection intensity (number of zoospores on skin swabs) and zoospore output (number of zoospores released per minute) at death.

Prior exposure	Total days survived post-inoculation	*Bd* infection intensity at death	Zoospore output at death
Naïve	21	520,436	3.5
Naïve	28	1,697,306	0.0
Naïve	18	4,454,759	4.9
Naïve	31	8,781,016	0.2
Naïve	25	9,584,158	170.6
Previous	18	2,291,631	7.1
Previous	33	2,960,916	0.0
Previous	31	4,385,154	0.0

**Figure 2:**
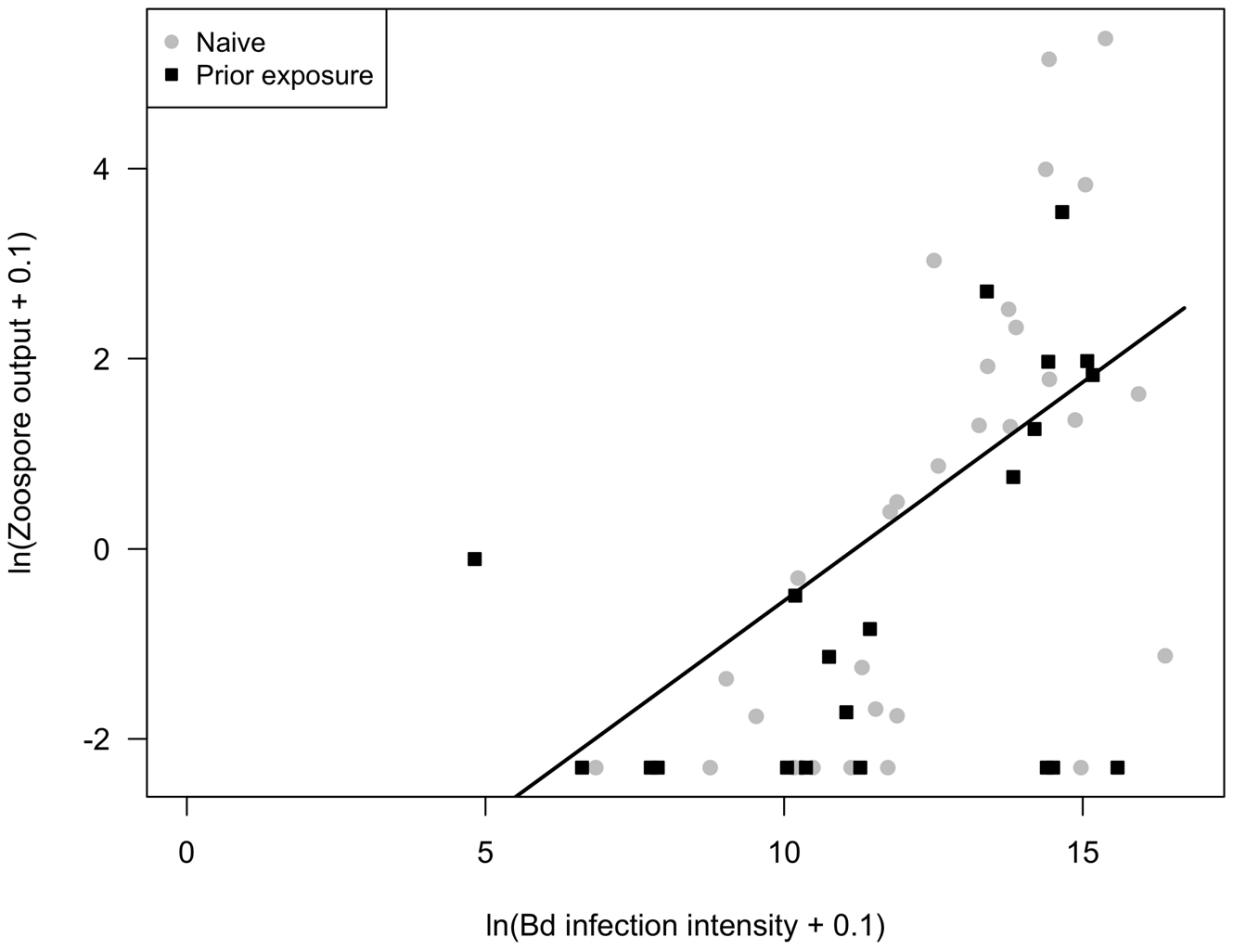
Relationship between *Bd* infection intensity and zoospore output. The solid black line corresponds to the linear regression fitted to all points (*t_43_* = 3.926, *p*<0.001). *Bd* infection intensity and zoospore output were positively correlated and not influenced by prior *Bd* exposure of the amphibian.

*Bd* infection intensity and zoospore output increased exponentially over time (*t_50_* = 7.018, *p*<0.001; *t_46_* = 3.164, *p* = 0.001, respectively). Including prior exposure or higher order polynomials did not improve model fit. The daily percent increase in *Bd* infection intensity and zoospore output were 35.4% (SE: 0.05) and 13.1% (SE: 0.04), respectively. *Bd* infection intensity and zoospore output were positively correlated (Figure 2; *t_43_* = 3.926, *p*<0.001). Prior *Bd* exposure did not affect *Bd* infection intensity or zoospore output (*t_6_* = 1.896, *p* = 0.106; *t_6_* = 0.624, *p* = 0.555, respectively). Survival rates also did not differ between naïve and previously exposed individuals (*p*>0.05).

Filtered water from frog soaks produced more false negatives than skin swabs. Seventeen soaks tested negative, even though skin swabs tested positive. Only three swabs tested negative during the entire experiment. At time of death, three individual soaks tested *Bd* negative, although swab infection intensity from the same sampling period was extremely high (Table 1), suggesting either zoospores were trapped in the filters or the PCR reaction was inhibited.

## Discussion

Exposing *Atelopus zeteki* to *Bd* strain JEL 423 produced individuals with *Bd* infection intensities among the highest reported for any species to date (Table 2). Individuals also had high zoospore output, indicating *A. zeteki* were highly infectious and may contribute disproportionately to the environmental *Bd* zoospore pool. Other experimental infections [26], [34] and field studies [35] also show that *Atelopus* spp. develop high *Bd* infection intensities, further suggesting that the genus *Atelopus* may be acute supershedders.

**Table 2:** Average *Bd* infection intensity of adult amphibians at death by several experimental studies.

Species	Study	*Bd* strain	Average *Bd* infection intensity at death
*Bufo boreas*	Carey et al. [22]	JEL 275*	10^7^ to 10^8^
*Atelopus zeteki*	Becker et al. [34]	JEL 408*	>10^6^
*Atelopus zeteki*	This study	JEL 423*	>10^6^
*Litoria booroolongensis*	Cashins et al. [47]	Native*	10^4^ to 10^5^
*Pseudacris regilla*	Reeder et al. [4]	Unknown	2.2×10^5^
*Atelopus zeteki*	Langhammer et al. [26]	JEL 427-P9	1.2×10^5^
*Atelopus zeteki*	Langhammer et al. [26]	JEL 427-P39	7.2×10^4^
*Rana sierrae*	Rosenblum et al. [48]	Sierra Nevada-*Bd* *	5.6×10^4^
*Rana muscosa*	Rosenblum et al. [48]	Sierra Nevada-*Bd* *	2.2×10^4^
*Rana muscosa/sierrae*	Stice and Briggs [36]	LJR119*	5.1×10^3^

* indicates the *Bd* strain used occurs within the amphibian species native range.

Other *Atelopus* studies have shown similarly high *Bd* infection intensities. Experimental infections of *A. zeteki* with other *Bd* strains (another Panamanian isolate JEL408 and a Puerto Rican isolate JEL427) showed *Bd* infection intensities ranging between 7.2×10^4^ and >10^6^ zoospores at death (Table 2; [26], [34]). Field studies also show high infection intensities in other species of *Atelopus*. Lampo et al. [35] reported the *Bd* infection intensity of a single dying *Atelous crucifer* individual as high as 244,000 zoospores. We cannot rule out *Bd* identity as the cause of variable high infection intensities at death because *Atelopus* were exposed to different *Bd* strains. Yet, the infection intensities in all lab and field studies were very high and caused rapid mortality.

Although we used an unnaturally high inoculation dose in this experiment, our results and conclusions are applicable to field scenarios because they mimic late stage infections. Carey et al. [22] showed that all individuals of *Bufo [Anaxyrus] boreas* died of infection at the same *Bd* infection intensity, those receiving lower doses only took longer to build infections and die. We used a high inoculation dose to minimize the duration of the experiment. Further studies are needed to document *Bd* infection intensities of *Atelopus* in the field and to determine whether *Atelopus* drives disease dynamics in other species.

We not only found that *Bd* infection intensity in *A. zeteki* at time of death was >10^6^, but that *A. zeteki* had a high daily rate of increase in *Bd* infection intensity and zoospore output. We are only aware of a few studies that have quantified the daily rate of increase in *Bd* infection intensity [22], [36] or zoospore output [28]. *Bufo [Anaxyrus] boreas* had daily percent increases in *Bd* infection intensity of 68% and produced individuals with >10^7^ zoospores at death (Table 2). Interestingly, *Rana [Lithobates] muscosa/sierra* had daily percent increases in *Bd* infection intensity of only 8% and infection intensities at death were approximately 10^4^ zoospores [36]. Meanwhile, *Litoria caerulae* had a daily rate of increase in zoospore output of 15.43% (SE: 2.29; [28]), but we were unable to compare the *Bd* infection intensity at death or mortality rate of this species to others because it was not reported. Yet, the first three species mentioned (*A. zeteki*, *B. boreas*, and *R. muscosa/sierra*) have experienced mass mortality and widespread population declines [6], [7], [24], [25], [37]–[39], suggesting that where infections build rapidly, frogs die with higher burdens.

Our study also provides evidence that *Bd* pre-exposure is insufficient to change the outcome of infection. This suggests that either (1) *A. zeteki* can not mount an effective adaptive immune response or (2) *Bd* possibly evades [40] and/or suppresses the immune system [41]–[43]. For example, Fites et al. [43] showed that *Bd* cells and supernatant impaired lymphocyte proliferation and induced apoptosis. The three individuals that were inoculated with JEL427-P39 may have persisted with mild infections during the first experiment because of several mechanisms acting singly or in concert: (1) their immune system was able to minimize infections, (2) the attenuated strain did not reproduce well, or (3) the inoculation was ineffective. We have no data to inform the first or second possibility, although the first possibility seems unlikely given the eventual mortality of those individuals; and the third possibility can be eliminated, given that all individuals, except one, tested *Bd* positive during the experiment.

*Ex situ* captive assurance *Atelopus* colonies are used as conservation tools to prevent extinction of the genus, with the ultimate goal of returning individuals to their native habitats. Yet, high *Bd* infection intensities and zoospore output of *A. zeteki* may create challenges for reintroduction programs. Not only do *Atelopus* experience high mortality rates when exposed to *Bd*, but there is substantial cause for concern if *Atelopus* are acute supershedders. To determine the feasibility of *Atelopus* reintroductions, future studies should examine *Bd* infection intensity, zoospore output, and immune function of *Atelopus* under different environmental conditions (e.g., [44]–[46]). Understanding infectivity, duration of infectiveness, and transmission heterogeneity among amphibian species and populations will lead to a more comprehensive understanding of factors leading to different disease outcomes among populations following *Bd* invasion.
